# Excess weight in employees of food and nutrition units at a university in São Paulo State

**DOI:** 10.1590/S1679-45082013000400014

**Published:** 2013

**Authors:** Juliano dos Santos, Aline Alves Ferreira, Karina Cardoso Meira, Angela Maria Geraldo Pierin

**Affiliations:** 1 Universidade de São Paulo, São Paulo, SP, Brazil.; 2 Universidade Federal do Rio de Janeiro, Rio de Janeiro, RJ, Brazil.; 3 Instituto Nacional de Câncer – INCA, Rio de Janeiro, RJ, Brazil.

**Keywords:** Overweight, Obesity, Nutrition personnel, Nutrition surveys, Occupational health

## Abstract

**Objective:**

To describe the prevalence and identify the factors associated with excess weight in restaurant employees at a public university in the city of São Paulo.

**Methods:**

A socioeconomic and nutritional census was conducted with 174 individuals to obtain data on body mass, height, and socioeconomic status, using a structured questionnaire. The body mass index was determined, and the cut-off points recommended by the World Health Organization were used. Student's *t* test, Fisher's exact test, and the χ^2^ test were used to verify the differences between the means and prevalences. Poisson regression analyses with robust variance were performed, and the outcomes were excess weight or no excess weight.

**Results:**

Most of the employees (57.5%) were women; 59.8% were non-white, 45.4% lived with a partner, 26.4% were smokers, and 50.6% were sedentary. There was a predominance of individuals with excess weight (60.9%), and most of them (64.0%) were women, non-white (66.3%), lived alone (58.8%), and were non-smokers (63.3%); furthermore, 62.8% of the subjects engaged in physical activities. There was a significant difference (p=0.03) regarding body mass index and gender, demonstrating more excess weight among the women. Excess weight was dependent on the age group and was more likely to occur in individuals over 50 years of age (adjusted prevalence ratio: 1.72; 95% confidence interval: 1.02 - 2.98).

**Conclusion:**

There was a high prevalence of excess weight in these professionals, indicating the necessity for interventions to control this important risk factor for chronic non-communicable diseases.

## RESUMO

**Objetivo:**

Descrever a prevalência e identificar os fatores associados ao excesso de peso em funcionários dos restaurantes de uma universidade pública da cidade de São Paulo.

**Métodos:**

Realizou-se um censo socioeconômico e nutricional com 174 indivíduos, por meio do qual foram coligidos dados referentes à massa corporal e à estatura, além de dados socioeconômicos, por meio de um questionário estruturado. Construiu-se o índice de massa corporal e utilizaramse os pontos de corte recomendados pela Organização Mundial da Saúde. Testes *t* de Student, exato de Fischer e χ^2^ foram utilizados na verificação das diferenças entre médias e prevalências. Análises de regressão de Poisson com variância robusta foram realizadas, sendo o excesso de peso ou não os desfechos.

**Resultados:**

A maioria dos funcionários (57,5%) era mulheres, 59,8% não brancos, 45,4% vivendo com o companheiro, 26,4% fumantes e 50,6% sedentários. Houve predomínio de indivíduos com excesso de peso (60,9%) que na maioria dos casos (64,0%) eram mulheres, não brancos (66,3%), viviam sozinhos (58,8%), não fumavam (63,3%) e (62,8%) praticavam atividade física. Observaram-se diferença significativa (p=0,03) em relação ao índice de massa corporal e ao gênero, evidenciando maior excesso de peso entre as mulheres. O excesso de peso foi dependente da faixa etária, havendo maior chance de ocorrer nos indivíduos com mais de 50 anos (razão de prevalência ajustada: 1,72; intervalo de confiança de 95%: 1,02–2,98).

**Conclusão:**

Observou-se alta prevalência de excesso de peso nesses profissionais, sinalizando a necessidade de intervenções para controle desse importante fator de risco para doenças crônicas não transmissíveis.

## INTRODUCTION

The health risks that workplaces may offer are a major burden to society in terms of morbidity and mortality, in addition to the financial and social costs^(1)^. On average, individuals spend one-quarter of their lives at work, and pressure related to time, environmental requirements, and the type of work performed can affect their eating habits and physical activity patterns, leading to overweight and obesity^(1,2)^.

Excess weight is currently a subject of global interest, and it is an important modifiable risk factor for several chronic diseases, such as cardiovascular disease.

In Brazil, there has been an increased frequency of overweight and obesity in all age groups, especially in the last 30 years, characterizing an accelerated nutrition transition process in the country^(3)^.

Among workers, especially those working at Food and Nutrition Units (FNU), this scenario is not different: overweight and obesity often affect more than 60% of this segment of the population^(4,5)^.

FNU workers depend on adequate workspace and equipment to perform their duties and to fulfill their roles in the workplace successfully. It is noteworthy that socioeconomic, cultural, and demographic factors can influence their nutritional status^(6)^.

The awareness of positive relationships between working conditions, health, and productivity has spurred investigations of this group of workers because studies have suggested an increase in chronic diseases, especially obesity^(2,7,8)^. This scenario has been associated with the nature of the job, and there is evidence of increased excess weight and changes in eating habits after workers commence this occupation^(8)^.

Recognizing FNU workers and understanding the potential issues associated with these changes in nutritional and food status is essential, as studies involving specific population strata have demonstrated that the prevalence of overweight / obesity vary depending on the region, the subgroup studied, or the diagnostic criteria used.

## OBJECTIVE

This study aimed to describe the prevalence and identify the risk factors associated with excess weight in restaurant employees at a public university in the city of São Paulo.

## METHODS

The present investigation was a cross-sectional study developed from a nutritional and socioeconomic census conducted from December 2005 to February 2006 with 174 individuals from a group of 180 employees working at six restaurants at the University of São Paulo (*Universidade de São Paulo* - USP), located in the city of São Paulo. Participant losses were related to absenteeism and holidays.

FNUs act as producers and distributors, with capacity to serve 16,350 meals per day, including breakfast, lunch, and dinner, and there are eight units in total^(9)^.

The eligibility criteria were to be employed at one of the restaurants for more than 6 months and to be actively working during the data collection period. Pregnant employees were excluded.

Using structured and self-reported interviews, the following data were collected: sociodemographic and economic data such as gender, age, race/color, marital status, education, and family income (in Brazilian *Reais*), as well as lifestyle habits such as smoking and physical activity.

For the nutritional assessment, the anthropometric variables used were weight and height, which were obtained according to the method of Lohman et al.^(10)^. To measure the body mass index (BMI), a digital scale (Health o Meter 752KL Buffalo Grove, Illinois, USA) with 150kg maximum capacity and 0.1kg precision was used. To measure height, a compact wall-mounted stadiometer (Sanny compact stadiometer, model ES: 2040/Sanny – American Medical do Brasil, Ltda, São Bernardo do Campo, Brazil), with 2m capacity and 0.1cm precision was used.

The BMI was obtained from the anthropometric measurements. The BMI values were analyzed according to the criteria defined by the World Health Organization (WHO)(^11^): normal weight (<25kg/m^2^), overweight (25 to 29.9kg/m^2^), and obesity (≥30kg/m^2^).

### Data processing and analysis

For data analysis, the variable age was categorized in years according to the age ranges (<30, 30 to 40, 40 to 50, and >50 years); race/color was classified as white (for those who reported having white skin) and non-white (for those who reported having brown or black skin). The marital status was classified as with a partner (married or common-law marriage) and without a partner (single, separated, divorced, or widowed); the household income was stratified into >3 monthly minimum wages and <3 monthly minimum wages; education was categorized according to years of study: <9 years, 9 - 12 years, and >12 years. The smoking habit was classified as yes (regularly smokes) and no (ex-smoker: cessation for ≥12 months, or never having smoked).

The assessment of physical activity was based on WHO recommendations(^12^) and on the consensus defined by the National Institutes of Health (NIH)^(13)^ and was categorized as yes (individuals with at least 30 minutes of continuous or accumulated physical activity per day) and no. Excess weight was defined as the sum of the overweight and obesity categories, which were obtained using the BMI.

The statistical analysis was developed in four stages: descriptive analysis, bivariate analysis, multivariate analysis, and residual analysis, using the statistical program R-2.7.1. In the descriptive analysis of the data, the mean, median, variance, and standard deviation (SD) were calculated for the quantitative variables, as well as the absolute (n) and relative (%) frequencies of the categorical variables. The next step involved the bivariate analysis to verify whether each exposure variable was associated with the response variable.

The relationship between categorical variables was assessed using the χ^2^ test or Fisher's exact test. For the quantitative variable (age), the difference between the means of overweight/obese individuals and normal individuals, and the difference between genders were assessed with Student's *t* test. In the multivariate analysis, for factors associated with excess weight, the prevalence ratio and the confidence intervals (95%CI) were calculated using the Poisson regression with robust variance, and the presence or absence of overweight was the outcome. In these analyses, the “sandwich” library of the statistical program R-2.7.1 was used.

The independent variables were gender, race/color, smoking habits, physical activity, marital status, age range, education, and income. The first five variables were used dichotomously, and the BMI was categorized as normal weight and excess weight (overweight/obesity). The covariates that demonstrated a critical level of p≤0.20 were considered candidates for the final model.

The adjustment of potential confounding variables was performed by using the multivariate technique in a stepwise manner, and the variables that were significantly associated according to the bivariate analysis were included in the final model. After all main effects were simultaneously included, the plausible interactions were tested.

The selection of the final model considered the residual analysis, by graphical observation and clinical and epidemiological significance. Values were considered significant at p≤0.05.

The study was approved by the Research Ethics Committee of the Nursing School of USP, process # 475/2005.

All employees who agreed to participate signed an informed consent form (ICF), in duplicate.

## RESULTS

Table 1 lists the characteristics of the sample. There was a predominance of female subjects, aged between 40 and 50 years, white, without a partner, with family incomes higher than 3 monthly minimum wages, and with 9 to 12 years of education.

**Table 1:** Distribution of employees (n=174) according to sociodemographic characteristics, lifestyle, and comorbidities. São Paulo, 2006

Variables		n (%)
Gender		
	Female	100 (57.5)
	Male	74 (42.5)
Age group		
	<30	18 (10.3)
	30–40	44 (25.3)
	40–50	72 (41.4)
	>50	40 (23.0)
Race/color		
	White	70 (40.2)
	Non-white	104 (59.8)
Marital status		
	With partner	79 (45.4)
	Without partner	95 (54.6)
Income		
	>3 monthly minimum wages	89 (51.1)
	<3 monthly minimum wages	85 (48.9)
Education		
	<9 years	6 (3.4)
	9–12 years	149 (85.6)
	>12 years	19 (10.9)
Smoking habits		
	Yes	46 (26.4)
	No	128 (73.6)
Physical activity		
	Yes	86 (49.4)
	No	88 (50.6)

The age of the studied population ranged from 21 to 65 years (mean 43.2 years, SD=9.7). The mean age of the women was 45.7 years (SD=8.7), whereas the mean age of the men was 39.7 years (SD=9.6). Notably, there was a significant difference between the genders (p=0.001) regarding the mean age. A significant difference (p=0.005) was also observed between the mean age of individuals with normal weight and individuals with excess weight.

The interviewees reported smoking (26.4%), and 49.4% performed physical activities.

Table 2 presents the prevalence of excess weight, characteristics of the individuals with excess weight, and associated factors.

**Table 2:** Relationship between the nutritional status and independent variables in restaurant employees at a public university in São Paulo State. São Paulo, 2006

Variables		Nutritional status					p value
		Excess weight		Normal weight		Total	
		n	%	n	%	n	
Gender*							
	Female	64	64.0	36	36.0	100	0.03
	Male	42	56.8	32	43.2	74	
Age range*							
	<30	8	44.4	10	55.6	18	
	30–40	24	54.5	20	45.5	44	0.09
	40–50	44	61.1	28	38.9	72	
	>50	30	75.0	10	25.0	40	
Race/color*							
	White	37	52.9	33	47.1	70	0.07
	Non-white	69	66.3	35	33.7	104	
Marital status*							
	With partner	51	48.1	55	51.9	106	0.37
	Without partner	40	58.8	28	41.2	68	
Smoking habits*							
	Yes	25	54.3	21	45.7	46	0.29
	No	81	63.3	47	36.7	128	
Income*							
	>3 monthly minimum wages	54	60.7	35	39.3	89	0.95
	<3 monthly minimum wages	52	61.2	33	38.8	85	
Physical activity*							
	Yes	54	62.8	32	37.2	86	0.62
	No	52	59.1	36	40.9	88	
Education**							
	<9 years	5	83.3	1	16.7	6	0.25
	9–12 years	57	38.3	92	61.7	149	
	>12 years	10	52.6	9	47.4	19	

* Two tailed *χ* ^2^ test, p≤0.05;

** Two-tailed Fisher's exact test, p≤0.05.

There was a predominance (60.9%) of individuals with excess weight, and most of them were non-white women, aged over 50 years, who lived without a partner, were nonsmokers, had an income of less than 3 monthly minimum wages, engaged in physical activity, and had less than 9 years of education. There was a significant difference (p=0.03) regarding BMI and gender, demonstrating more excess weight among the women. In addition, there was a higher concentration of overweight/obesity in the older individuals.

The variables gender (p=0.34), smoking habits (p=0.31), physical activity (p=0.61), income (p=0.94), and marital status (p=0.36) were not significant at 20%, and were excluded from the multivariate analysis (data not shown in the tables).

Table 3 lists the variables included in the multivariate analysis.

**Table 3:** Unadjusted and adjusted prevalence ratio for factors associated with excess weight in restaurant employees at a public university in São Paulo State. São Paulo, 2006

Variables		Overweight/obesity			
		n	%	Unadjusted PR	Adjusted PR
Age range					
	<30	8	7.5	1	1
	30–40	24	22.6	1.21 (0.68 - 2.16)	1.37 (0.77 - 1.44)
	40–50	44	41.5	1.40 (0.80 - 2.42)	1.50 (0.88 - 1.44)
	>50	30	28.3	1.68 (0.98 - 2.91)	1.72 (1.02 - 2.98)
Education					
	<9 years	5	6.9	1	1
	9–12 years	57	79.1	0.74 (0.50 - 1.78)	0.60 (0.38 - 1.30)
	>12 years	10	13.8	0.56 (0.31 - 1.02)	0.87 (0.61 - 1.24)
Race/color					
	White	37	34.9	1	1
	Non-white	69	65.9	1.26 (0.97 - 1.63)	1.25 (0.97 - 1.62)

PR: prevalence ratio.

Only the age group variable remained associated with overweight/obesity.

Individuals aged over 50 years had a greater chance of being overweight compared with the younger individuals (adjusted PR: 1.72, 95% CI=1.02–2.98).

## DISCUSSION

The present study revealed that, in the population of FNU workers studied, 36.8% were overweight, and 24.1% were obese (data not shown), totaling 60.9% individuals with excess body weight. This frequency is higher than the findings of the latest Household Budget Survey (HBS) conducted by the Brazilian Institute of Geography and Statistics (*Instituto Brasileiro de Geografia e Estatística* - IBGE) between 2008 and 2009, in which 12.4% of male adults and 16.9% of female adults were obese^(3)^. However, this same survey demonstrated overweight in more than one-half of the Brazilian population, with values similar those found in workers in the present study: 62.5% among men and 64.9% among women^(3)^.

In a national telephone survey conducted in the same year in which data for the present study were collected, the prevalence of overweight was 42.7%, and the prevalence of obesity was 11.4%^(14)^. These frequencies have been changing rapidly over the past decades. In the 1970s, the percentage of the Brazilian population exhibiting excess weight was 22.2% (1974/1975). In the following decade, this value increased to 39.1% (1989), reaching 47.0% in 1995/1996^(15)^. Other studies in different Brazilian states have also revealed a similar trend^(16,17)^.

Although only a few investigations have examined the nutritional status of FNU employees, these studies have also revealed a significant prevalence of excess weight^(6,8,18,19)^.

Among employees of a university restaurant in Rio Grande do Sul, 74.6% gained an average of 2.6 kg during the first year on the job^(20)^. In American FNU employees, the frequency of obesity increased from 7.1% in 1986 to 12.0% in 1995 for males and from 8.5% to 15.3% for females^(7)^. Nonetheless, these results are far below the values observed in the present study.

Obesity is an important risk factor for several diseases, especially hypertension and diabetes. Obesity is considered a chronic disease, and along with other chronic diseases, it is responsible for over 70% of the deaths in Brazil regardless of socioeconomic status^(21)^.

Women are most often affected by overweight and obesity, as shown by different studies with employees of restaurants and kitchens^(16,20,22)^. According to Boclin and Blank^(18,19)^, the nature of the work and the distribution of tasks can be an important factor in this difference. Men tend to participate in activities that involve greater physical effort, and they are less exposed to foods and food consumption. Although the increase of overweight and obesity in men is significant^(16,22)^, this gender difference has also been found in other Brazilian studies^(3,16)^.

Excess weight has increased in both genders, with the most significant impact on women of lower socioeconomic status^(21)^. In the present study, no socioeconomic variable was significantly associated with excess weight, which can be partially explained by the fact that the population is homogeneous (with very similar activities) and in a single sector.

However, the highest prevalence of overweight was found in people who lived alone, were not white, smoked, had fewer years of education, and were female.

The association of low education with overweight was also evident in the technical and administrative staff of a university in Rio de Janeiro^(23)^.

In employees benefited by the Workers Food Program (*Programa de Alimentação do Trabalhador* - PAT) in São Paulo, the years of study were a protective factor against excess weight^(24)^. In non-white workers from different sectors, the prevalence of overweight was also higher, which agreed with the findings of the present study^(8)^.

The smoking and family arrangement were not associated with obesity in any study with workers. Some studies have revealed a positive relationship between smoking and BMI^(16,25)^. The presence of family members in the household may be related to disease prevention, greater financial stability, and health promotion^(26)^. Lifestyle has a direct influence on the nutritional status of the individual^(27)^. Thus, the incidence of excess weight tends to be higher in individuals who live alone, in agreement with the present study.

Despite the relatively young age profile of the evaluated population, age was the only variable significantly associated with excess weight. With advancing age, the likelihood of being overweight increases by 5% compared with younger people, and age has been consistently associated with a higher prevalence of overweight and obesity, regardless of gender^(14,28)^.

According to the Surveillance of Risk and Protective Factors for Chronic Diseases by Telephone (VIGITEL), which was conducted in 2010, the prevalence of obesity in men is almost 4 times higher in the age range from 45 to 54 years compared with younger people (18 - 24 years). In women, the values triple according to the age, declining only in women over 65 years^(14)^.

In workers at 30 companies registered in PAT, in the city of São Paulo, age was considered a risk factor for excess weight, regardless of gender^(24)^.

The lack of association with other variables can corroborate the high influence that occupational characteristics may have on the health and nutrition status of individuals.

The proximity and excessive exposure to food can stimulate FNU employees to consume more^(6,18)^. High consumption between meals – namely, “snacks” and “samples”, especially of sugary drinks and fatty foods – may be a possible explanation^(20)^.

The foods that FNU workers eat have been characterized as high-calorie foods, and there is a high consumption of foods rich in protein and fat^(6)^.

The requirement for a greater consumption of food to maintain FNU employees' work activities, which require physical effort, may also be an explanation. Moreover, the opportunity to eat foods that are not usually consumed at home can contribute to the scenarios reported herein.

However, the lack of association between overweight and the other variables, except for age, may be related to the relative homogeneity among these FNU workers.

There are certain limitations to the present study. Because this was an exploratory investigation, food consumption was not analyzed in depth in this first instance. The population was specific, which prevents generalizations, and it comprised only FNU employees in the city of São Paulo, excluding employees at FNUs on *campuses* away from the capital, which might have masked some of the differences. Such limitations should be addressed in future studies.

## CONCLUSIONS

Excess weight is a common problem. However, the nature of many interactions between the workplace and healthy weight maintenance is not well studied or understood, and this situation has yielded limited interventions directed at promoting health in professionals working in FNUs.

A high prevalence of excess weight was observed, and age was an important factor associated with overweight and obesity.

These findings indicate that maintaining a proper weight should be a priority. Educational initiatives that are based on holistic and interdisciplinary points of view and that are directed at promoting health and healthy eating practices are essential for this group.
